# A Topic Modeling Comparison Between LDA, NMF, Top2Vec, and BERTopic to Demystify Twitter Posts

**DOI:** 10.3389/fsoc.2022.886498

**Published:** 2022-05-06

**Authors:** Roman Egger, Joanne Yu

**Affiliations:** ^1^Innovation and Management in Tourism, Salzburg University of Applied Sciences, Salzburg, Austria; ^2^Department of Tourism and Service Management, Modul University Vienna, Vienna, Austria

**Keywords:** topic model, machine learning, LDA, Top2Vec, BERTopic, NMF, Twitter, covid travel

## Abstract

The richness of social media data has opened a new avenue for social science research to gain insights into human behaviors and experiences. In particular, emerging data-driven approaches relying on topic models provide entirely new perspectives on interpreting social phenomena. However, the short, text-heavy, and unstructured nature of social media content often leads to methodological challenges in both data collection and analysis. In order to bridge the developing field of computational science and empirical social research, this study aims to evaluate the performance of four topic modeling techniques; namely latent Dirichlet allocation (LDA), non-negative matrix factorization (NMF), Top2Vec, and BERTopic. In view of the interplay between human relations and digital media, this research takes Twitter posts as the reference point and assesses the performance of different algorithms concerning their strengths and weaknesses in a social science context. Based on certain details during the analytical procedures and on quality issues, this research sheds light on the efficacy of using BERTopic and NMF to analyze Twitter data.

## Introduction

With its limitless availability of constantly growing datasets and simultaneous increase in computing power, the era of digital transformation has brought about the potential to alter social science (Lazer and Radford, 2017). These massive volumes of data assemble digital footprints and capture cumulative human activities, both individually and collectively (Boccia Artieri et al., 2021). As such, the rise of big data in the twenty-first century has prompted a demand for advanced analytic techniques such as machine learning, natural language processing (NLP), and topic modeling in order to uncover patterns and relations embedded in the data, reduce the dimensionality of data, and forecast future outcomes more effectively (Elragal and Klischewski, 2017). In particular, the use of topic modeling in social science [e.g., conventional models such as Dirichlet allocation (LDA) and non-negative matrix factorization (NMF)] has soared in popularity across various domains in the past years (Maier et al., 2018; Chen et al., 2019). These techniques rely on statistical modeling to extract topical patterns within a collection of texts (Egger and Yu, 2021). For instance, since a semantic relationship exists between terms like “apple,” “pear,” and “mango,” they could be formed under a topic called “fruit” in a text corpus (i.e., a collection of documents). Typically, documents contain mixed membership, which means that a mixture of topics exists in the corpus (Maier et al., 2018).

To unfold the complex nature of social phenomena, topic models act as a bridge between social science and (un)structured analysis, different methods of reasoning, and big data analytics (Hannigan et al., 2019) due to their explorative character (Albalawi et al., 2020). In social science, implications of big data can range from macro-level analyses (e.g., social structure and human behavior) to micro-level analyses (e.g., individual relationships and aspects of daily activities). Based on observed phenomena and experiences, examples can be noted from a growing amount of literature analyzing the news (Chen et al., 2019), online reviews (Bi et al., 2019), and social media content (Yu and Egger, 2021), amongst others. Yet, while the discussion of big data in social science mainly circles around the critical perspective of the subject, the application itself is hardly ever deliberated. Although big data seems exceptionally promising, data is always preconfigured through beliefs and values, and numerous challenges must be acknowledged as every step in big data analysis depends on various decisive criteria, such as the selection of parameters, the evaluation of partial results, and the actual interpretations thereof (Lupton, 2015). With recent advancement in the NLP field, emerging modeling techniques such as BERTopic (Grootendorst, 2022) and Top2Vec (Angelov, 2020) further complicate the process of big data analytics, pressing the need to evaluate the performance of different algorithms. Additionally, while social scientists are interested in theory-based assumptions and their implications, data scientists focus on discovering new patterns (Cai and Zhou, 2016) that appear to be irrational due to their limited explanatory power for social phenomena (McFarland et al., 2016).

Social media has opened an entirely new path for social science research, especially when it comes to the overlap between human relations and technology. In this respect, the value of user-generated content on social media platforms has been well-established and acknowledged since their rich and subjective information allows for favorable computational analysis (Hu, 2012). For instance, recent research explored the social dynamics of sporting events based on Facebook comments (Moreau et al., 2021), while another study disclosed the social semiotics of different attractions using Instagram content (Arefieva et al., 2021). Scholars have also used Twitter posts related to the COVID-19 pandemic to construct individual's reactions (Boccia Artieri et al., 2021). From an epistemological viewpoint, what is common among these data-driven approaches is that they provide brand-new perspectives on interpreting a phenomenon and have the possibility to revamp state-of-the-art knowledge (Simsek et al., 2019). After all, many aspects of social science and social media intertwine in one way or another; while the former concerns human interaction, the latter escalates its essence to a much broader and global scale.

Nevertheless, despite the prominence of social media in today's society, posts are often text-heavy and unstructured, thereby complicating the process of data analysis (Egger and Yu, 2021). Such methodological challenges are particularly salient for those lacking programming knowledge and skills (Kraska et al., 2013). Certainly, recent advancements in visual programming software have enabled researchers to analyze social media data in a coding-free manner using topic modeling (Yu and Egger, 2021), yet the validity and quality of the findings based on such intuition remain questionable. One common misconception that may skew results is the use of default hyperparameter settings. Although the importance of model tuning has been frequently acknowledged (Zhou et al., 2017), little guidance can be found when analyzing social media data in social science. In addition, another barrier that hinders knowledge generation in social science contexts is the application of more traditional and commonly-adopted algorithms (Blair et al., 2020). For example, despite the popularity of LDA, the reliability and validity of results have been criticized since model evaluation is left behind (Egger and Yu, 2021).

Consequently, some social scientists have initiated a call to conduct more interdisciplinary research and evaluate model performance based on other new and emerging techniques (Reisenbichler and Reutterer, 2019; Albalawi et al., 2020; Egger and Yu, 2021). Appertaining to the insufficient knowledge of newly developed algorithms that could better handle the nature of social media data in social science, this study thus aims to evaluate and compare the performance of four topic modeling techniques, namely, LDA, NMF, Top2Vec, and BERTopic. Specifically, LDA is a generative statistical model, NMF uses a linear algebra approach for topic extraction, and BERTopic and Top2Vec use an embedding approach. By bridging the discipline of data science with social science, reviews of the strengths, and weaknesses of different tools are valuable to support applied social scientists in choosing appropriate methods. This research sheds light on the capabilities of alternative solutions that can facilitate social science scholars in coping with any methodological issues when addressing big data.

## Literature Review

### Making Sense of Social Media Using Machine Learning Models

With the omnipresent use of technologies, human communication has transcended time and space, both locally and globally (Joubert and Costas, 2019). Among the various types of communication tools, social media stands out as a vital medium in mediating and facilitating interactions between social actors (Murthy, 2012). As social media portrays human behavior and interactions, social scientists have proceeded with data mining (Boccia Artieri et al., 2021) and using NLP and machine learning approaches. In order to understand the vast numbers of posts shared on social media, NLP can comprehend human languages, as programmed for machines, to make predictions based on the observed social phenomena (Hannigan et al., 2019). On the other side, machine learning, as a part of artificial intelligence, refers to computational methods using existing databases (i.e., the training data) to build and train a model for prediction and better decision making (Zhou et al., 2017). The advantages of opening new horizons for sociological consideration through advanced data analytics can be witnessed in manifold contexts, including business, healthcare, education, and, more generally, the role of social activities in developing scientific knowledge (Yang et al., 2020).

Previous research has underlined that the digital revolution presents dynamics in exchange networks (Joubert and Costas, 2019) and implies one's self-perception (Murthy, 2012). Examples can be seen from microblogging sites such as Twitter, accumulating over 200 million daily active users. As social media transforms interactions into relationships, and those interactions evolve into experiences (Witkemper et al., 2012), continuous status updates are seen and valued as self-production (Murthy, 2012) and, thus, allow scientists to assess perspectives from the public's point of view (Joubert and Costas, 2019). For instance, in infodemiology, Xue et al. (2020) applied machine learning models to monitor public responses in relation to the COVID-19 discussion and concerns on Twitter. Likewise, in the highly-dynamic tourism industry, Lu and Zheng (2021) were able to track public opinions toward cruise ships during the COVID-19 pandemic based on collected tweets. Furthermore, unlike most networking platforms built upon existing friendships, the retweet function can disseminate information much faster (Park et al., 2016), thereby making Twitter an ideal medium for social science research.

Yet, regardless of which social media platform, theorization remains an integral part (Müller et al., 2016) of the emerging subject of big data in social science. Although some scholars believe that big data can, and should, be free of theory altogether (Anderson, 2008; Kitchin, 2014), it seems improbable to interpret results without a sufficient understanding of the social sciences (Mazanec, 2020). Nevertheless, methodological challenges often present themselves in parallel with epistemological developments. For instance, because algorithms are unable to structure free text, data preprocessing steps that require complex decision-making skills, such as cleaning, transformation, feature extraction, and vectorization, lay the foundation for further analysis (Albalawi et al., 2020). Though social scientists have the ability to preprocess the datasets, issues may arise in the following steps involving model evaluation and hyperparameter tuning (Blair et al., 2020). For the most part, these challenges can be traced back to the nature of social media content itself, which primarily consists of short, concise, text-heavy, and unstructured formats (Albalawi et al., 2020).

### Topic Modeling as a Solution to Cope With Unstructured Text Data

As human language is an adaptive multilevel system, text length, syntactic complexity, and semantic plausibility have long been considered focal points in both psychology and linguistics (Bradley and Meeds, 2002). Together with the interplay between technology and modernization, their impact has also extended to social media. For instance, scholars have pointed out that shorter posts typically lead to a higher engagement rate on Facebook (Sabate et al., 2014), potentially because concise messages reduce the amount of cognitive effort needed for information processing (She et al., 2022). Across the various available types of platforms, Twitter, in particular, restricts each post to a maximum of 280 characters (Queiroz, 2018), and although these short and unstructured posts conform with social media practice, they increase the complexity for algorithms to make sense of digital interaction. Common challenges arise from using compound words, acronyms, and ungrammatical sentences (Ariffin and Tiun, 2020). Despite the productive and unexpressed nature of compound words they often complicate computational analysis (Krishna et al., 2016). Other difficulties emerge when data are meaningless (i.e., noisy data) or when there are many gaps present in the data (i.e., sparse data; Kasperiuniene et al., 2020).

In order to effectively extract features from a large corpus of text data, numerous text mining approaches have been introduced (Li et al., 2019), among which topic modeling serves as the most frequently adopted technique (Hong and Davison, 2010). In a nutshell, a topic model is a form of statistical modeling used in machine learning and NLP, as discussed earlier, that identifies hidden topical patterns within a collection of texts (Guo et al., 2017). Those viewed as the most established, go-to techniques include LDA, latent semantic analysis (LSA), and probabilistic LSA (Albalawi et al., 2020). More recently, however, newly developed algorithms such as NMF, Corex, Top2Vec, and BERTopic have also received, and are continuing to attract, increasing attention from researchers (Obadimu et al., 2019; Sánchez-Franco and Rey-Moreno, 2022). In the social sciences, topic models have formerly been applied to, for example, discover consumers' implicit preferences (Vu et al., 2019; Egger et al., 2022), identify semantic structures on Instagram (Egger and Yu, 2021), and improve recommendation systems (Shafqat and Byun, 2020). Despite the robustness of topic modeling algorithms, existing literature relies primarily on one single model, with LDA being the dominant method (Gallagher et al., 2017) and is typically viewed as the standard approach.

Regardless of the popularity of LDA within the social science branch, its efficacy in analyzing social media data has been highly criticized (Egger and Yu, 2021; Sánchez-Franco and Rey-Moreno, 2022). In the case of Twitter data, Jaradat and Matskin (2019) argue that, while multiple topics can coexist in a document, LDA tends to neglect co-occurrence relations. Likewise, other researchers emphasize that noisy and sparse datasets are unsuitable for LDA (Chen et al., 2019) due to a lack of features for statistical learning (Cai et al., 2018). Consequently, researchers have reinforced the value of newly developed algorithms as alternatives since they often outperform LDA, especially when analyzing short text data on social media (Egger, 2022b). Albeit new approaches have emerged and have been adopted to reveal novel insights, their innovative advantages (unintentionally) lower the significance of model evaluation. Evidence can be taken from social media research, to which applying evaluation techniques is yet to become mainstream (Reisenbichler and Reutterer, 2019). Furthermore, because models would be optimized in extracting any slight variant of a topic, depending on the purpose of the algorithm, the results might be skewed in a specific direction. These issues further highlight the unreliability of concentrating solely on one single topic model and, thereby, also strengthening the value and need to compare differing algorithms (Reisenbichler and Reutterer, 2019; Albalawi et al., 2020; Egger and Yu, 2021).

## Materials and Methods

Intrigued by the complexity of short-text social media data, the goal of this research is to compare different types of topic modeling algorithms in order to offer new insights and solutions to social scientists interested in investigating human interactions. Compared to other platforms, Twitter features concise posts, with a maximum of 280 characters per tweet, that can be identified *via* specific hashtags (Queiroz, 2018). The use of hashtags thus streamlines the information search process based on users' interests. Seeing the potential of social media in enhancing crisis communication (Femenia-Serra et al., 2022), this study makes use of Twitter posts related to travel and the COVID-19 pandemic as reference points for the evaluation of the four above-mentioned topic models (i.e., LDA, NMF, Top2Vec, and BERTopic). The detailed implementation process of this study proceeded as below.

### Data Collection and Preprocessing

Data collection was conducted in November 2021 by using the data extraction software tool Phantombuster and searching for the terms #covidtravel as well as the combination of #covid and #travel to fetch tweets. The initial datasets included a total of 50,000 tweets posted in English; however, after cleaning the data and removing duplicate posts, the final datasets consisted of 31,800 unique tweets. After that, the data underwent preprocessing in which all mentions (e.g., ^@^users), hashtags, unknown signs, and emojis were removed. It is important to note that, up to this point, original sentences were used for BERTopic and Top2Vec since both algorithms rely on an embedding approach, and keeping the original structure of the text is vital for transformer models.

On the other hand, the data for LDA and NMF was preprocessed further using NLP modules in Python. More precisely, stopwords were excluded, irrelevant text (e.g., numbers, abbreviations, and unknown characters) was removed, and tokenization was performed. Following this step, stemming and lemmatization were then conducted. The former process used Porter Stemmer to remove suffixes from words (e.g., investigating to investigate), whereas the latter used WordNet Lemmatizer to remove inflectional endings and to return a word to its base form (e.g., investigating to investigate). Lastly, the text was converted into term frequency-inverse document frequency (TF-IDF) weight for information retrieval based on the importance of a keyword.

### Implementation of Topic Models

#### Model 1: Latent Dirichlet Allocation

LDA, the most popular topic modeling technique, is a generative probabilistic model for discrete datasets such as text corpora (Blair et al., 2020). It is considered a three-level hierarchical Bayesian model, where each collection item is represented as a finite mixture over an underlying set of topics, and each topic is represented as an infinite mixture over a collection of topic probabilities. Hence, as the number of topics need not be pre-defined (Maier et al., 2018), applying LDA provides researchers with an efficient resource to obtain an explicit representation of a document.

**Figure 1:**
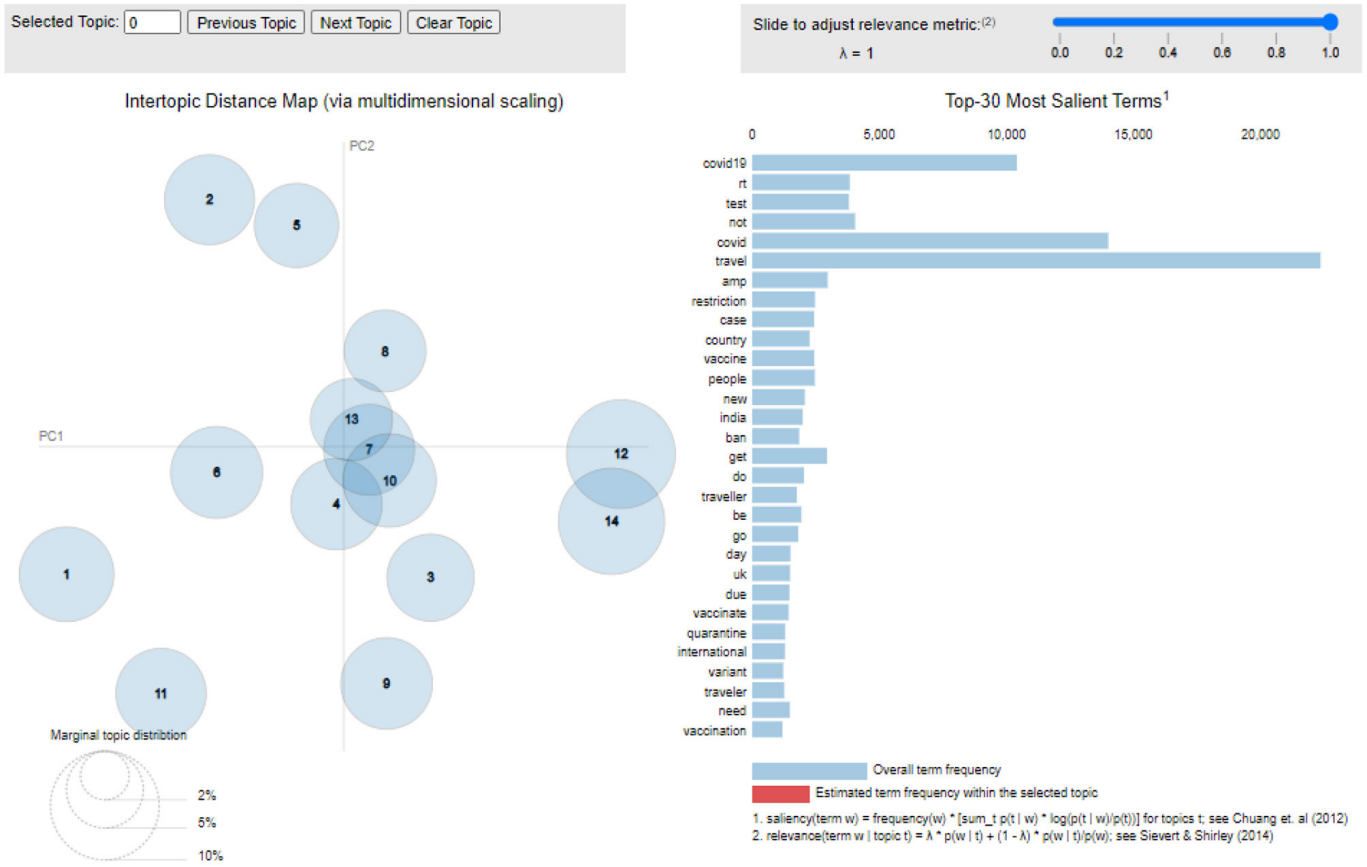
Visual inspection of LDA.

In this research, to pinpoint optimal values for the three hyperparameters required for LDA, a grid search was performed for the number of topics (*K*) as well as for beta and alpha. The higher the beta, the more words the topics consist of; likewise, the higher the alpha, the more diverse the topics are. The search for an optimal number of topic*s* started with a range from two to 15, with a step of one. In the first step of the learning process, *K* was pre-defined, and the search for beta and alpha was applied accordingly. During the process, only one hyperparameter varied, and the other remained unchanged until reaching the highest coherence score. The coherence score, referring to the quality of the extracted topics, presented itself for 14 topics with a value of 0.52. The grid search then yielded a symmetric distribution with a value of 0.91 for both alpha and beta. Finally, to facilitate a clear interpretation of the extracted information from a fitted LDA topic model, pyLDAvis was used to generate an intertropical distance map (Islam, 2019). A screenshot of the statistical proximity of the topics can be seen in Figure 1. An interactive visualization is available at https://tinyurl.com/frontiers-TM.

#### Model 2: Non-negative Matrix Factorization

**Figure 2:**
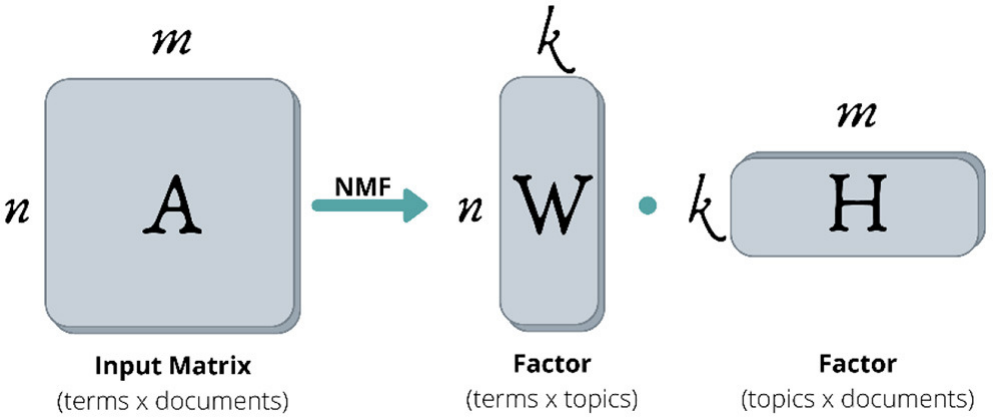
Intuition of NMF. Source: Egger (2022b).

In contrast to LDA, NMF is a decompositional, non-probabilistic algorithm using matrix factorization and belongs to the group of linear-algebraic algorithms (Egger, 2022b). NMF works on TF-IDF transformed data by breaking down a matrix into two lower-ranking matrices (Obadimu et al., 2019). Specifically, TF-IDF is a measure to evaluate the importance of a word in a collection of documents. As demonstrated in Figure 2, NMF decomposes its input, which is a term-document matrix (*A*), into a product of a terms-topics matrix (*W*) and a topics-documents matrix (*H*) (Chen et al., 2019). The values of *W* and *H* are modified iteratively, where the former contains the basis vectors, and the latter contains the corresponding weights (Chen et al., 2019). It is necessary that all entries of *W* and *H* are non-negative; otherwise, the interpretation of topics with negative values would be difficult (Lee and Seung, 1999).

Since NMF requires the data to be preprocessed, necessary steps to be performed beforehand include a classical NLP pipeline containing, amongst others, lowercasing, stopword removal, lemmatizing or stemming as well as punctuation and number removal (Egger, 2022b). For this study, an open-source Python library, Gensim, was used (Islam, 2019) to estimate the optimal number of topics. By computing the highest coherence score, 10 topics could be identified.

#### Model 3: Top2Vec

Top2Vec (Angelov, 2020) is a comparatively new algorithm that uses word embeddings. That is, the vectorization of text data makes it possible to locate semantically similar words, sentences, or documents within spatial proximity (Egger, 2022a). For example, words like “mom” and “dad” should be closer than words like “mom” and “apple.” In this study, a pretrained embedding models, the Universal Sentence Encoder, was used to create word and document embeddings. Since word vectors that emerge closest to the document vectors seem to best describe the topic of the document, the number of documents that can be grouped together represents the number of topics (Hendry et al., 2021).

However, since the vector space usually tends to be sparse (including mostly zero values), a dimension reduction was performed before density clustering. By using uniform manifold approximation and projection (UMAP), the dimensions were reduced to the extent that hierarchical density-based spatial clustering of applications with noise (HDBSCAN) could be used to identify dense regions in the documents (Angelov, 2020). Finally, the centroid of the document vectors in the original dimension was calculated for each dense area, corresponding to the topic vector.

Notably, because words that appear in multiple documents cannot be assigned to one single document, they were recognized by HDBSCAN as noise. Therefore, Top2Vec does not require any preprocessing (e.g., stopwords removal), or stemming and lemmatization (Ma et al., 2021; Thielmann et al., 2021). To conclude this model, Top2Vec automatically provided information on the number of topics, topic size, and words representing the topics.

#### Model 4: BERTopic

BERTopic (Grootendorst, 2020) builds upon the mechanisms of Top2Vec; hence, they are similar in terms of algorithmic structure. As the name suggests, BERT is used as an embedder, and BERTopic provides document embedding extraction, with a sentence-transformers model for more than 50 languages. Similarly, BERTopic also supports UMAP for dimension reduction and HDBSCAN for document clustering. The main difference between Top2Vec is the application of a class-based term frequency inverse document frequency (c-TF-IDF) algorithm, which compares the importance of terms within a cluster and creates term representation (Sánchez-Franco and Rey-Moreno, 2022). This means that the higher the value is for a term, the more representative it is of its topic.

**Figure 3:**
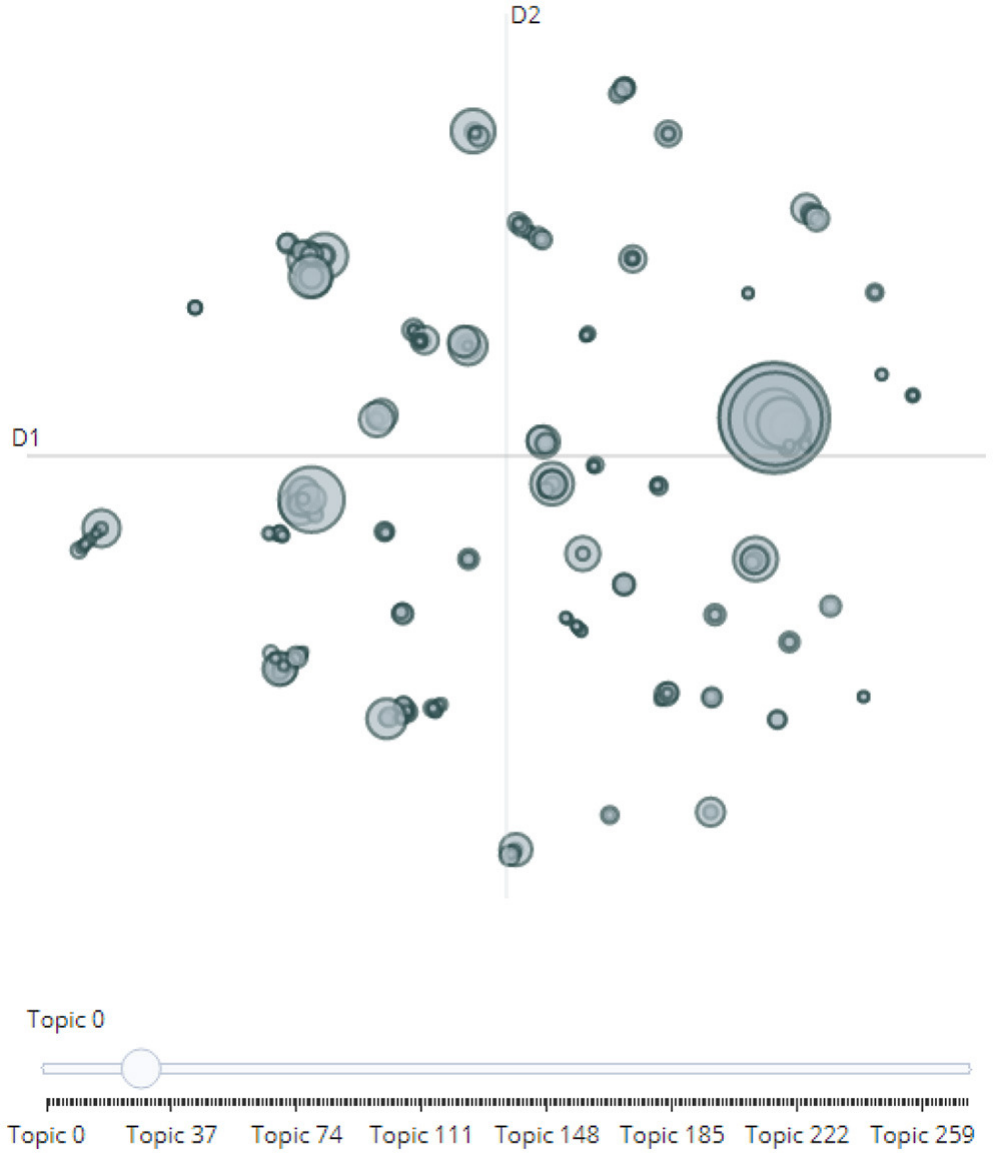
BERTopic's interactive intertopic distance map.

BERTopic, similar to Top2Vec, differs from LDA because it provides continuous rather than discrete topic modeling (Alcoforado et al., 2022). The stochastic nature of the model thus leads to different results with repeated modeling. Once the model is computed, researchers can output the most important topics. Notably, Topic 0 with a count of−1 will always represent outliers and should not be considered any further. Researchers can also search for a keyword and receive the most important topics based on their similarity score along with the possibility to inspect individual topics based on their keywords. Ultimately, in order to better analyze the potentially large array of topics, BERTopic offers an interactive intertopic distance map for inspecting individual topics (Grootendorst, 2020). As illustrated in Figure 3, once an initial overview of the topics becomes available, an automated topic reduction can be performed again.

## Results

In essence, although topic models bring in statistical analysis and can advance social science research, each of the algorithms has its own uniqueness and relies on different assumptions. Quantitative methods are limited in their ability to provide in-depth contextual understanding, and the results cannot be compared with any single “value” (Egger and Yu, 2021). Thus, the interpretation of models still relies heavily on human judgment (Hannigan et al., 2019) and researchers' domain knowledge (Egger and Yu, 2022).

In the following section, a comparison of the obtained results will be divided into two parts, according to the nature of the algorithm: (1) LDA and NMF and (2) Top2Vec and BERTopic. The latter highlights the term search function as one of the pros of using a guided/seeding approach to delve deeper into a specific topic.

### Comparison of LDA and NMF

Table 1 provides an overview of the 14 identified topics in the LDA model and the 10 topics from NMF. Names were given based on the terms that contributed the most to a topic in reference to their TF-IDF weights. Overall, several aspects point to common themes, such as expectations toward government response, discussion on *R*_(t)_ values, and travel restrictions in different countries. Taking “government response” as an example, tweets seem to focus on people's expectations toward the White House (e.g., #whcovidresponse) and the US president (#potus, #vp). Although both models refer to the chance to reunite with their loved ones (e.g., #loveisnottourism), LDA, in particular, points out how the COVID-19 pandemic has influenced the Diversity Visa Program (e.g., #dv2021) application. Likewise, while both models disclose Twitter users' opinions on travel ban restrictions and quarantine, the LDA results appear to be more geographically oriented. For instance, when discussing the reproduction number, European countries, India, and the UK are more frequently mentioned. On the other hand, England and Scotland appear to be the main focal point concerning travel restrictions, and as for tweets related to quarantine, LDA reveals issues surrounding the Australian border.

**Table 1:** Topics identified by LDA and NMF.

	**LDA**		**NMF**	
**No**.	**Topic/content**	**Keywords**	**Topic/content**	**Keywords**
1	Government response	ban, travelgov, potus, dv2021, loveisnottourism, whcovidresponse, end, visa, please, vp	Government response	whcovidresponse, potus, loveisnottourism, cdcdirector, presssec, vp, cdctravel, cdcgov, liftthetravelban, cdctravel cdcdirector
2	Association for Molecular Pathology (AMP) / mask and virus	amp, travel, come, spread, mask, place, follow, stay, keep, virus	Association for Molecular Pathology (AMP) / desire to travel	covid, travel, people, amp, want, covid travel, time, travel covid, like, year
3	R_t_ value / India, UK, Europe	rt, travel, country, India, uk, covid, government, list, eu, news	R_t_ value	rt, covid, travel, https, covid19, traveler, rt ollysmithtravel, traveler, httpstco, ollysmithtravel
4	Travel restriction / England and Scotland	travel, covid, restriction, city, team, England, despite, event, expect, Scotland	Travel restriction	restriction, travel restriction, covid travel, covid19 travel, ease, covid restriction, travel, lift, covid19 restriction, restriction lift
5	Vaccination / border between Canada and the USA	vaccinate, covid19, international, traveler, travel, vaccination, Canada, border, US, fully	Travel ban / India and UK	ban, India, travel ban, travel India, uk, list, country, ban travel, red, variant
6	Quarantine and lockdown / Australia	traveler, day, quarantine, variant, allow, return, lockdown, Australia, break, two	General about travel / Canada	covid19, travel, covid19 travel, international, travel covid19, country, pandemic, international travel, vaccination, Canada
7	COVID-19 cases / USA	case, new, travel, health, state, tourism, public, number, close, include	Vaccination and quarantine	vaccinate, fully, fully vaccinate, vaccinate covid19, traveler, vaccinate traveler, traveler, quarantine, cdc, require
8	Flight / COVID-19 test	test, travel, need, positive, covid, flight, negative, air, take, airport	COVID-19 cases / New Zealand	case, new, covid case, covid19 case, new case, rise, Zealand, New Zealand, report, case covid19
9	Death / Florida	covid, die, death, cause, florida, child, spike, shoot, traveler002, flu	COVID-19 test	test, covid test, negative, positive, test travel, test positive, PCR, covid19 test, day, result
10	China and USA	travel, covid, call, china, business, 2020, trump, usa, dr	Vaccination pass	vaccine, covid19 vaccine, covid vaccine, passport, vaccine passport, require, vaccine travel, dose, mandate, vaccination
11	Unspecific I	not, covid, vaccine, people, do, travel, get, make, still, would		
12	Unspecific II	travel, may, covid, 2, please, 1, help, show, 3, pass		
13	Unspecific III	covid19, travel, due, pandemic, world, today, first, update, coronavirus, safe		
14	Unspecific IV	covid, be, go, travel, time, get, want, one, year, see		

Still, in spite of LDA performing seemingly better up to this point, the model produces more universal and irrelevant topics that, at the same time, barely offer any meaningful implications. This can be evidenced from the final four LDA topics listed in Table 1, which, based on the keywords, center on travel and COVID-19 on a broader level. Therefore, despite the fact that only a few NMF topics contain country-specific terms (e.g., New Zealand, India, and the UK), its value should not be underestimated. Due to a clear distinction between all the identified topics in the NMF model, this research concludes that the results obtained from NMF are more in line with human judgment, thereby outperforming LDA in general. Yet, as mentioned above, since topic extraction with LDA and NMF relies primarily on hyperparameters, most of the results are within expectation. As both models, however, do not allow for an in-depth understanding of the phenomenon, the next section will focus on the topic models that use embedding representations.

### Comparison of BERTopic and Top2Vec

By relying on an embedding model, BERTopic and Top2Vec require an interactive process for topic inspection. As such, both algorithms allow researchers to discover highly relevant topics revolving around a specific term for a more in-depth understanding. Using Top2Vec for demonstration purposes, this section begins with the intuition behind the search query. Presuming that there is an interest in topics related to the term “cancel” during COVID-19, the Top2Vec model produces relevant outputs (topics) based on the order of their cosine similarity (Ghasiya and Okamura, 2021). Specifically, cosine similarity, ranging from 0 to 1, measures the similarity between the search term and a topic. In the case of this research, out of 309 topics, the similarity of Topic 10 proved to be the highest [*0.50*], followed by Topic 20 [*0.37*], Topic 7 [*0.33*], Topic 123 [*0.32*], and Topic 57 [*0.30*].

Thereafter, the most important keywords for each individual topic can be retrieved. For example, the keywords for Topic 10 include the following:

*[“refund,” “booked,” “ticket,” “cancelled,” “tickets,” “booking,” “cancel,” “flight,” “my,” “hi,” “trip,” “phone,” “email,” “myself,” “hello,” “couldn,” “pls,” “having,” “guys,” “am,” “sir,” “supposed,” “hopefully,” “me,” “excited,” “postpone,” “so,” “days,” “dad,” “paid,” “option,” “customers,” “request,” “bihar,” “thanks,” “amount,” “due,” “waiting,” “to,” “got,” “back,” “impossible,” “service,” “hours,” “complete,” “before,” “wait,” “nice,” “valid,” “book”]*.

**Figure 4:**
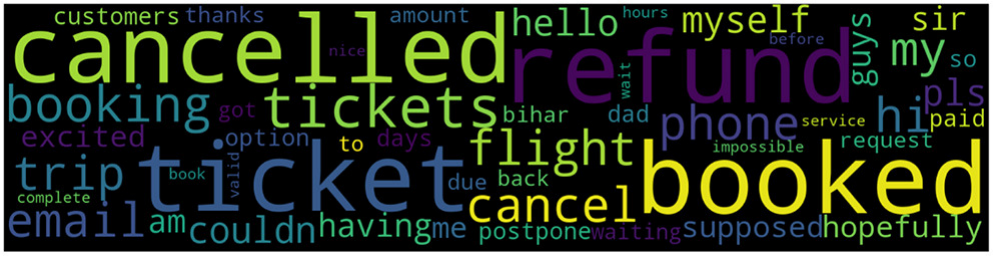
Example of a word cloud based on the term “cancel.”

In order to acquire an overview of the importance of each term, a word cloud can be produced for better visualization (see Figure 4); but, ultimately, an inspection of individual tweets is also highly recommended. For instance, the findings suggest that document 20189 (tweets: “@*PaytmTravel Flight - AI 380 dated 9th April, 2020 (Canceled due to COVID). No Refund since then […]*”) has a similarity score of *0.8518*. This information allows one to gain deeper insights directly from the raw data. Meanwhile, in order to find more suitable keywords based on “cancel” for even further analysis, words that are most similar can be output with their similarity, such as “canceled [*0.60*],” “refund [*0.49*],” “booked [*0.47*],” “due [*0.46*],” and “ticket [*0.43*].”

Following the search process, a topic comparison between Top2Vec and BERTopic could be established. This time, “flight” and “travel bubble” were taken as other examples. Since cosine similarity has previously been introduced, the following section merely lists some of the keywords that facilitate topic naming. As mentioned above, this is because the results require human interpretation to make sense of the data (Hannigan et al., 2019).

Starting with “flight,” Table 2 provides an overview, out of the 343 identified topics, of the six most relevant ones taken from BERTopic and five, out of 253, from Top2Vec. Overall, Top2Vec topics appear to be more policy- and regulation-oriented, focusing on pre-departure testing requirements (e.g., negative PCR test and full vaccination) in countries such as Mexico, the Netherlands, and Canada. It also discusses the government's travel advice for public transport, such as in trains, buses, and flights. For a more qualitative inspection, relevant tweets can be reviewed; take, for example, “*Kind attention dear passengers traveling to […] Please follow COVID-19 norms at the airport. Fly safe!*” and “*[My] flight [got] canceled by airlines due to covid. Also my travel insurance premium wasted*.” On the other hand, topics identified by BERTopic are more related to the nature of air transport. Specifically, common issues shared on Twitter include the airline industry, flight routes, returning home, transmission through air, and air travel associations.

**Table 2:** Topics identified by BERTopic and Top2Vec for “flight.”

	**BERTopic**		**Top2Vec**	
**No**.	**Topic/content**	**Examples of keywords**	**Topic/content**	**Examples of keywords**
1	Airline industry	air travel, airline, air travel is, airlines, aviation, flights, the airline industry, the airline, airline industry, flight	Negative PCR / vaccination and quarantine	hours before, pre-departure, negative covid, all travelers, fully vaccinated, pcr, quarantine, days, requirement, mandatory
2	Flight routes	flights from, flights, direct flights, flights from india, canada eyes policy, canada eyes, india to canada, to canada, ban on direct, as india covid19	White House Secretary Tests Positive / travel guide from governmental institution	secretary, simon, house, white, tested positive, travel guidelines, cdc, mps, travelers, to follow
3	(Unable) to return home / Australian	australians, travel ban, fly home, fly home from, who fly home, who fly, to australia, australians who fly, covid travel ban, travel ban	Negative PCR / fully vaccinated before departure / foreign travelers / Mexico	negative covid, fully vaccinated, foreign travelers, pre departure, hours before, required to, before you, to enter, pcr, mexico
4	COVID transmission through air	the air, aerosols, droplets, air, airborne, covid travels, through the air, virus travels, how covid travels, covid travels through	Negative PCR / fully vaccinated before departure / foreign travelers / the Netherlands and Canada	negative covid, departure, hours before, international travelers, fully vaccinated, biden, the united, requirement, netherlands, canadians
5	Airports Authority of India (AAI) / India	aai, airports, aai airports, airport, the airport, flights, aai is, airports are, from aai, air traffic	Follow travel guidelines on public transport (train / bus / flight) / seek help and more info	train, bus, while traveling, covid appropriate, more information, to follow, covid guidelines, mandatory, by air, please help
6	Airport news	news airport airtravel, airtravel covid19 covid19india, airport airtravel, airport airtravel covid19, travelers news airport, airtravel covid19, travel covid19, flight travel covid19, air travel associations, airports air	

Turning to “travel bubble,” both algorithms produced five relevant topics, as presented in Table 3. In this case, the BERTopic results seem to be more specific, with a clear distinction on travel between Australia and New Zealand, Singapore and Hong Kong, as well as Canada and Mexico. Other issues center on travel passes and business travel. With regards to Top2Vec, however, the results revealed a slight overlap. For example, the travel bubble between Australia and New Zealand is covered in four out of five topics; similarly, Singapore, Hong Kong, and Taiwan are also mentioned several times. In addition, Top2Vec produces topics with multiple aspects, which becomes especially apparent in the third and fourth topics. The third topic contains issues related to six different countries (i.e., Hong Kong, Singapore, Australia, New Zealand, the UK, and the Philippines), and the fourth includes quarantine regulations in eight countries (i.e., Singapore, Australia, New Zealand, Taiwan, Hong Kong, Korea, Hawaii, and Indonesia).

**Table 3:** Topics identified by BERTopic and Top2Vec for “travel bubble.”

	**BERTopic**		**Top2Vec**	
**No**.	**Topic/content**	**Examples of keywords**	**Topic/content**	**Examples of keywords**
1	Australia and New Zealand	travel bubble, travel bubble with, the travel bubble, australia travel bubble, zealandaustralia travel bubble, new zealandaustralia travel, zealand travel, zealand travel bubble, bubble with australia, after travel bubble	Australia and New Zealand / quarantine hotel	sydney, victoria, queensland, australia, hotel quarantine, nz, in hotel, quarantine free, lockdown, auckland
2	Singapore and Hong Kong	bubble, travel bubble, singapore, air travel bubble, travel bubble is, bubble is, singaporehong kong air, singaporehong kong, breaking singaporehong kong, as singapore battles	Australia and New Zealand / Singapore / Taiwan / vaccinated	zealand, quarantine free, singapore, hotel quarantine, 2 weeks, isolate, vaccinated travelers, lockdown, melbourne, Taiwan
3	Travel pass	travel pass, covid travel pass, eus covid travel, eus covid, the eus covid, covid travel, summer travel, travel passes, travel passes as, launch covid travel	Hong Kong and Singapore / Australia and New Zealand / green list / vaccinated / UK / Philippines	hong kong, singapore, zero covid, taiwan, green list, australia, vaccinated travelers, philippines, zealand, business travel
4	Nonessential travel / Canada and Mexico ferry / spread of COVID-19	canada and mexico, on non-essential travel, nonessential travel at, nonessential travel, ferry crossings, crossings with canada, ferry crossings with, land and ferry, and ferry crossings, spread of covid19	Quarantine free / Singapore / Australia and New Zealand / Taiwan / Hong Kong / Korea / Hawaii / Indonesia	quarantine free, singapore, hk, auckland, taiwan, korea, sydney, hawaii, indonesia, vaccinated travelers
5	Business travel	business travel, tourism, travel industry, the travel industry, tourism industry, and tourism, travel and tourism, and tourism industry, travel and, tourism industry the	Singapore / Hong Kong / Australia / Taiwan / fully vaccinated / green list	taiwan, singapore, hong kong, business travel, zealand, australia, fully vaccinated, portugal, green list, israel

As a final note, when inspecting the keywords of BERTopic and Top2Vec, despite the redundancy of some terms (e.g., “travel bubble” and “travelbubble,” as they are very close in the same vector-space), they can, in fact, provide valuable insights, especially for the process of topic naming. Mostly, the content of a topic can be understood based on frequently-repeated keywords. Moreover, regarding the logic of the algorithm, since BERTopic and Top2Vec should not be preprocessed, conjunction words (e.g., after, before to, from, at) are helpful for connecting the context. However, a major drawback without preprocessing is that (in)definite articles or be-verbs appearing in the keywords lists are often meaningless in comprehending a topic.

### Hierarchical Topic Reduction of Top2Vec and BERTopic

Finally, it is worth noting that both Top2Vec and BERTopic allow for hierarchical reduction. Echoing this study's results, the number of extracted topics tends to be relatively large, thereby necessitating the need for intensive qualitative analysis. In order to streamline the analysis, the algorithms offer the possibility to reduce these topics further (Angelov, 2020). Starting with Top2Vec, a hierarchical reduction down to 10 topics is typically considered a good starting point to begin topic analysis. In the case of this research, the 10 remaining clusters deducted from the 253 original topics are presented in Table 4. Significantly, the original vectors remain after topic reduction, meaning that representative topics with keywords can still be sought after at any time.

**Table 4:** Hierarchical topic reduction of Top2Vec.

**No**.	**Topic/content**	**Examples of keywords**
1	Diversity visa / Student life	byron, selectees fault, bay, mask, are increasing, student, the flu, exams, forever, first wave, take, traveling, covid positive, there, hands, rapidly, want, big, stop, death, interstate, fucking, haven, market, transmission, covid appropriate, bihar, to wear, short, exam, increasing
2	Diversity visa and visa petition / freedom / international travel / COVID-19 curfew	the petition, sign, tests for, pcr covid, selectees fault, boris, ford, ontario, want, curfew, premier, the airport, free, friend, trudeau, postpone, check out, rapidly, pakistan, shot, uk, enjoy, stay at, true, thread, toronto, travel insurance, international travel, normal, many countries, variants, overseas travel, freedom, mps, interstate, red list, folks, canadians, reasons, province, bihar
3	Diversity visa / unvaccinated people / vaccinate to prevent	selectees fault, centers for, di, disease, white, labor, fauci, economy, behavior, million, not being, market, shame, europeans, kerala, americans, control, here are, millions of, trump, unvaccinated, buy, weekend, make sure, oct, and tourism, dv, jobs, to protect, shop, this weekend, of vaccination, concerns, for your, air travel, next month, vaccines, open, to ease, political, millions, virus, prevention, cover, plans to, science, mexico, tourism
4	Politicians (Grant Shapps, Justin Trudeau, Biden, Trump, Anthony Fauci) / green list countries / international travel for vaccinated people / olympics / COVID-19 passport	on vaccination, eu, covid certificate, requirement for, ban, borders to, biden, grant, shapps, president, even worse, chinese, olympics, trudeau, european, required for, digital, vaccinated travelers, fauci, many countries, justice, vaccinated travelers, travel pass, visas, other countries, trump, the federal, countries, australians, green list, law, infected, joe, the border, for fully, interstate travel, europe, open, next month, covid passports
5	Pre-COVID and first wave / dreaming of travel	first wave, shelby, battle, solutions, simon, they find, the emergence, their journey, countless, lives, future, someone, human, money, an excuse, traveling, love, before covid, dose, happy, traveled, pfizer, from china, dream, together, selectees fault, died of
6	Complaints toward the US Diversity Visa Lottery program (COVID-19 as an excuse for the delay or cancellation thereof)	an excuse, toolset, selectees fault, even worse, on vaccination, uganda, death, justice, pcr tests, new cases, arabia, interview, the highest, united states, fun, winners, crazy, for fully, for foreign, nepal, imple, clear, african, nigeria, business travel, puerto rico, brexit, the airport, requiring, singapore
7	Yellow fever and COVID-19 vaccine / Saudi Arabia / COVID-19 cases	saudi, astrazeneca, journey, arabia, stay safe, new cases, covid numbers, dose of, nhs covid, wave, wear mask, got covid, yellow fever, pass, app, pre covid, doctors, eastern
8	Travel Destinations / Prevention / Travel Measures	dv, selectees fault, blaming, lanka, covid appropriate, rapidly, european, solutions, union, they find, the emergence, winners, travel advisory, increase, nepal, prevention, the delta, travel measures, covid cases, shelby, surge in, level, do not, new cases, travel related, eu, probably, hawaii, postpone, indian, to restrict, battle, florida, are increasing, rising covid, olympics, governor
9	Negative PCR test prior to departure / fully vaccinated for international travel	proof of, departure, hours before, covid appropriate, as long, will need, covid testing, negative covid, be fully, pre departure, to show, requirement for, you must, required to, by air, foreign travelers, test for, covid test, behavior, vaccinated against, test, pcr test, pcr tests, arrival, fully vaccinated, on vaccination, requirement, of vaccination, negative test, pcr, vaccination, negative, are fully, cdc, required, for international, requirements for, distancing, to require, guidance, on arrival, days of
10	Travel bubble / Australia (several cities included) and New Zealand / Hong Kong / Scotland / quarantine free / quarantine hotel	nsw, queensland, sydney, victoria, have tested, coast, shelby, melbourne, travel bubble, zealand, quarantine free, australia, in hotel, positive for, simon, wales, traveled from, kong, covid case, positive covid, battle, tested positive, first wave, vic, greater, auckland, woman, their journey, byron, the petition, hotel quarantine, scotland, south, army

**Figure 5:**
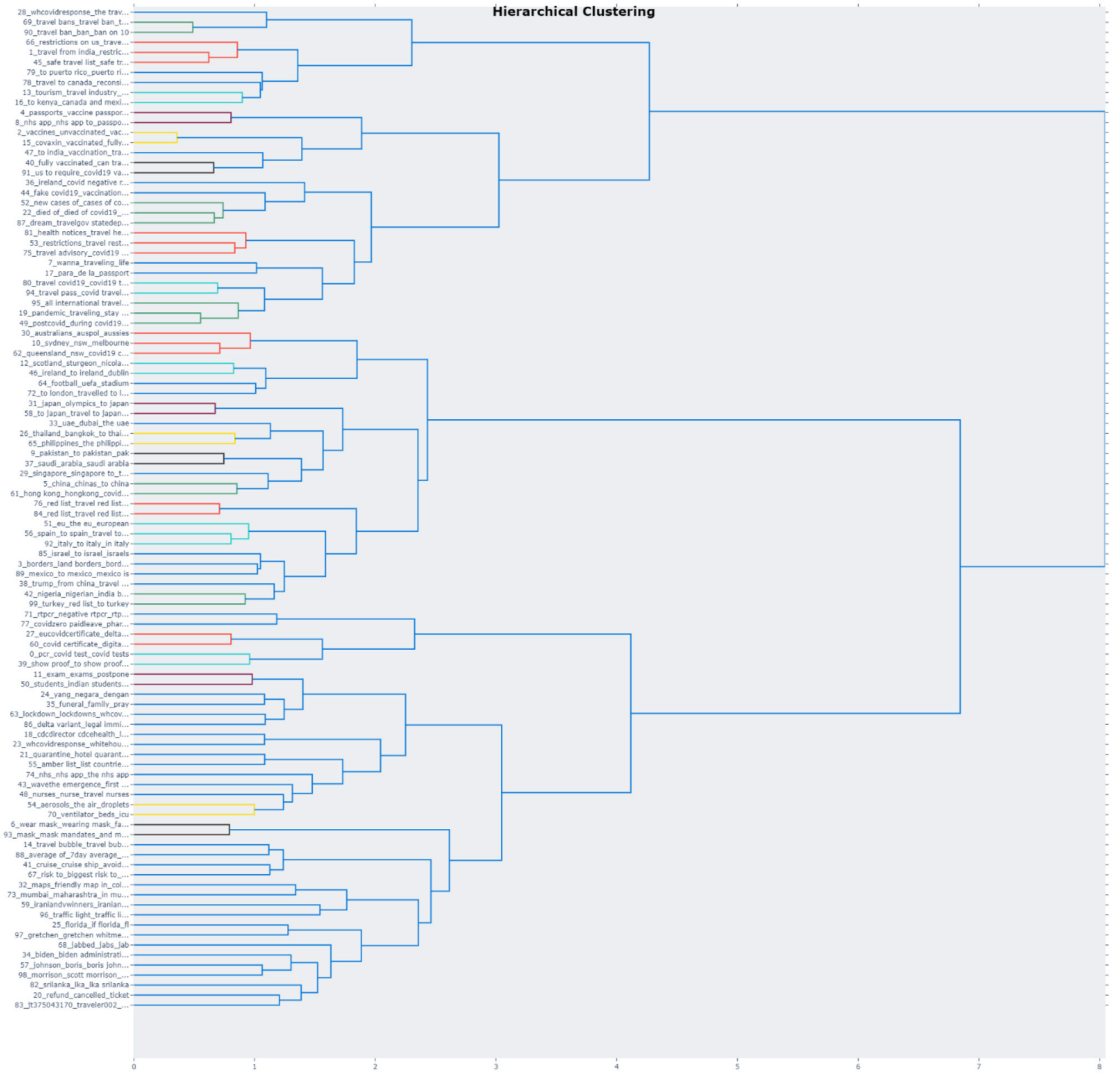
Hierarchical reduction in BERTopic.

Turning to BERTopic, since some of the topics are close in proximity, as could be observed in the intertopic distance map (Figure 3), visualization and topic reduction would provide a better understanding of how the topics truly relate to each other. To reduce the number of topics, hierarchical clustering was performed based on the cosine distance matrix between topic embeddings. This study thus took 100 topics as an example to provide an overview of how and to which extent topics can be reduced (Figure 5). Level 0 of the dendrogram demonstrates how similar topics (those with the same colors) have been clustered together. For example, Topic 4 (vaccine passports) and Topic 8 (the NHS COVID-19 app) were grouped together because of their adjacency. Correspondingly, Topic 6 (wearing face masks) and Topic 96 (mask mandate) were treated as part of the same cluster. In essence, a visualization as such can help researchers to better comprehend the algorithm's criteria by which topics are organized. After reviewing the proposed topic structure, researchers can then decide on a number of topics that also seem to be more realistic in an interactive manner.

However, for both algorithms, the underlying meanings of the topics are still subject to human interpretation. Nevertheless, although the intuition is to provide the best possible results, an optimal number of topics could not be established because most of the topics overlap with one another and cover a mixture of two to three different aspects. For instance, the results from Top2Vec (Table 4) present five topics associated with the US Diversity Visa program (e.g., dv, selectees fault, winners, an excuse, justice, interview, the petition, exam) and several terms related to politicians based in the USA and Canada (e.g., Grant Shapps, Justin Trudeau, Joe Biden, Donald Trump, Anthony Fauci). Similarly, making sense of the hierarchical clustering produced by BERTopic (Figure 5) also requires an enormous effort since the topic structure changes whenever researchers experiment with a different number of topics. Despite the possibility of using existing domain know-how to search for specific topics, a feature that is inexistent in other traditional algorithms, researchers should be well aware of the aforementioned issues. The overall process contains errors, and it may be quite labor-intensive to find a number that fits human judgment.

As shown in Figure 5 below, the dendrogram produced by BERTopic shows the agglomeration levels of the individual topics. This visualization, in particular, aids in finding an appropriate number of k-topics. Furthermore, similar to Top2Vec, a table with keywords is obtained after fusing the topics; yet, it is also highly recommended to inspect individual raw documents for more appropriate interpretations.

## Discussion and Conclusion

Baring the difficulties of extracting useful information from short and unstructured texts in mind, this research intends to confront such challenges by comparing the results of four topic modeling algorithms. For an overall evaluation based on human interpretation, this study supports the potency of BERTopic and NMF, followed by Top2Vec and LDA, in analyzing Twitter data. While, in general, both BERTopic and NMF provide a clear cut between any identified topics, the results obtained from NMF can still be considered relatively “standard.” Contrarily, in addition to the expected outcomes (i.e., topics), BERTopic was able to generate novel insights using its embedding approach. Although Top2Vec also uses pretrained embedding models, the results cover more topics that overlap and contain multiple concepts. On the other side of the spectrum, similar to NMF, the topics produced by LDA do not seem to be very intriguing, either. Thus, despite some Top2Vec topics appearing as irrelevant and difficult to understand, the model, even so, is capable of producing a few interesting findings rarely mentioned by other algorithms (e.g., politicians). As a result, in favor of extracting novel conclusions, this research recommends Top2Vec over LDA. To provide a more solid foundation for these reasonings, a detailed evaluation for each algorithm will now be given.

First and foremost, compared to other techniques, BERTopic works exceptionally with pretrained embeddings (Sánchez-Franco and Rey-Moreno, 2022) due to a split between clustering the documents and using c-TF-IDF to extract topic representations. Especially owing to the c-TF-IDF procedure (Abuzayed and Al-Khalifa, 2021), BERTopic can support several topic modeling variations, such as guided topic modeling, dynamic topic modeling, or class-based topic modeling. Its main strength lies in the fact that the algorithm performs well on most aspects of the topic modeling domain, whereas others typically excel in one single aspect. Additionally, after having trained a BERTopic model, it is also possible to reduce the number of topics (Sánchez-Franco and Rey-Moreno, 2022), subsequently allowing researchers to settle on a number of (realistic) topics based on how many were actually produced.

Slightly different from BERTopic and the implementation of c-TF-IDF, Top2Vec creates jointly embedded word, document, and topic vectors to find topic descriptions (Angelov, 2020). The intuition behind this algorithm is that every input is considered a vector, and pivoting between them is trivial. Hence, Top2Vec can scale a large number of topics and vast quantities of data. Such strength is especially required when multiple languages emerge within a corpus (Hendry et al., 2021). The main disadvantage of Top2Vec, however, is that it is unqualified to work with a small amount of data (Abuzayed and Al-Khalifa, 2021; e.g., <1,000 documents). In fact, BERTopic and Top2Vec have a number of issues in common. For example, although outlier generation might be beneficial in some cases, the solutions might actually generate more outliers than expected. Meanwhile, another flaw involves topic distributions: they cannot be generated within a single document because each document is assigned to a single topic. Although probabilities can indeed be extracted, they are not equivalent to an actual topic distribution.

With regards to NMF and LDA, notwithstanding that both algorithms do not require social scientists to have prior domain knowledge, several topics identified by LDA in this study yielded either universal (Rizvi et al., 2019) or irrelevant (Alnusyan et al., 2020) pieces of information. Such an issue further reflects the study's findings of LDA being indeterministic (Egger and Yu, 2021). In order to achieve optimal results, LDA usually requires detailed assumptions concerning the hyperparameters; in particular, discovering the optimal number of topics typically proves to be a difficult task (Egger and Yu, 2021). Although NMF shares the same disadvantages, it can be assumed that NMF puts forward better results since the algorithm relies on TF-IDF weighting rather than raw word frequencies (Albalawi et al., 2020). Simultaneously, as a linear-algebraic model, scholars commonly agree that NMF works well with shorter texts (Chen et al., 2019), such as tweets. Since no prior knowledge is needed for topic extraction (Albalawi et al., 2020), this strength specifically benefits research based on social media data (Blair et al., 2020). Additionally, as LDA extracts independent topics from word distributions, topics that are deemed dissimilar in the document may not be identified separately (Campbell et al., 2015), thereby resulting in overlapping clusters (Passos et al., 2011). In opposition, other scholars believe that insufficient statistical information for feature extraction is the fundamental factor behind duplicate topics (Cai et al., 2018).

Lastly, when comparing BERTopic to NMF, a major shortcoming of NMF revolves around its low capability to identify embedded meanings within a corpus (Blair et al., 2020). Considering that the algorithm depends primarily on the Frobenius norm (Chen et al., 2019), which is typically useful for numerical linear algebra, this issue ultimately leads to difficulties in interpreting findings (Wang and Zhang, 2021). Though NMF can effectively analyze noisy data (Blair et al., 2020), others argue that accuracy cannot be guaranteed (Albalawi et al., 2020).

Based on the outcomes of this study, as discussed above, Table 5 summarizes the pros and cons of applying LDA, NMF, BERTopic, and Top2Vec in order to help facilitate social scientists in the necessary preprocessing steps, proper hyperparameter tuning, and comprehensible evaluation of their results. However, researchers should take into account that, depending on the nature of the datasets, topic models may not always perform in the same fashion (Egger and Yu, 2021).

**Table 5:** Comparison of topic models.

	**Advantages**	**Disadvantages**
LDA	• Prior domain knowledge is not necessarily required • Finds coherent topics when correct hyperparameter tuning is applied • Can deal with sparse input • The number of topics is generally smaller than word-embedding based approaches; thus, it is easier to be interpreted • One document can contain several different topics (Mixed membership extraction) • Full generative models with multinominal distribution over topics are generated • Shows both adjectives and nouns within topics	• Detailed assumptions are required • Hyperparameters need to be tuned carefully • Results can easily produce overlapping topics as topics are soft clusters • Objective evaluation metrics are widely missing • The number of topics needs to be defined by the user(s) • Since the results are not deterministic, reliability and validity are not automatically ensured • Assumes that the topics are independent of each other; hence, only the frequency of the common occurrence of words is used • Word correlations are ignored, so no relationships between topics can be modeled
NMF	• Prior domain knowledge is not required • Supports mixed membership models; thus, one document can contain several topics • In contrast to LDA, which uses raw word frequencies, the term-document matrix can be weighted with TF-IDF • It proves to be computationally efficient and very scalable • Easy to implement	• Frequently delivers incoherent topics • The number of topics to be extracted must be defined by the user in advance • Implicit specification of probabilistic generative models
Top2Vec	• Supports hierarchical topic reduction • Allows for multilingual analysis • Automatically finds the number of topics • Creates jointly embedded word, document, and topic vectors • Contains built-in search functions (easy to go from topic to documents, search topics, etc.) • Can work on very large dataset sizes • It uses embeddings, so no preprocessing of the original data is needed	• The embedding approach might result in too many topics, requiring labor-intensive inspection of each topic • Generates many outliers • Not very suitable for small datasets (<1,000) • Each document is assigned to one topic • Objective evaluation metrics are missing
BERTopic	• High versatility and stability across domains • Allows for multilingual analysis • Supports topic modeling variations (guided topic modeling, dynamic topic modeling, or class-based topic modeling) • It uses embeddings, so no preprocessing of the original data is needed • Automatically finds the number of topics • Supports hierarchical topic reduction • Contains built-in search functions (easy to go from topic to documents, search topics, etc.) • Broader support of embedding models than Top2Vec	• The embedding approach might result in too many topics, requiring labor-intensive inspection of each topic • Generates many outliers • No topic distributions are generated within a single document; rather, each document is assigned to a single topic • Objective evaluation metrics are missing

### Theoretical and Practical Contributions

In light of the expansion of user-generated content, social media has broadened the horizons for human interaction and provoked new phenomena and social research for further investigation (Murthy, 2012; Rizvi et al., 2019; Boccia Artieri et al., 2021). Although several recent studies have vouched for the exploration of short-text social media data (Albalawi et al., 2020; Qiang et al., 2020), existing knowledge is rather restricted to conventional modeling techniques such as LDA and LSA (Albalawi et al., 2020). As the evolution of topic modeling has given rise to novel techniques, especially ones that have rarely been applied or evaluated in social science, this study is valuable in that it answers the call to assess topic modeling *via* a thorough comparison of four different algorithms (Reisenbichler and Reutterer, 2019). In addition, this research scrutinizes the bright and dark sides of applying embedded vs. standard topic models, but it also offers social science researchers insights into methodological challenges that may hinder knowledge generation.

Foreseeing that social scientists may indeed hesitate to choose an appropriate algorithm when analyzing social media data, this study presents possible methodological issues and promotes the efficacy of two different types of topic models. To be more precise, applying BERTopic to generate insights from short and unstructured text offers the most potential when it comes to embedding-based topic models. Thus, this study acknowledges the capability of BERTopic to encode contextual information (Chong and Chen, 2021), an aspect that may remain concealed by other models. Regarding traditional topic model algorithms, social science research is encouraged to consider NMF as an alternative approach to the commonly-adopted LDA (Gallagher et al., 2017). Certainly, however, it is essential to note that each model has its own strengths and shortcomings, and the findings require intensive qualitative interpretation. Finally, this study also strives to make another important contribution by outlining guided modeling solutions that can be applied by social scientists to data analytics for knowledge extraction.

### Limitations and Recommendations for Future Research

This research is certainly not without its limitations. While this study responds to a need to utilize Top2Vec and BERTopic for the analysis of short-text data (Egger and Yu, 2021; Sánchez-Franco and Rey-Moreno, 2022), novel language models, such as GPT3 and WuDao 2.0, have continued to emerge as time passes (Nagisetty, 2021), thereby acting as an excellent basis for even more powerful topic modeling approaches. To leverage the use of topic modeling methods, social scientists are encouraged to try and evaluate other newly developed algorithms and to keep their knowledge up to date. In the case of this study, Twitter was selected due to its strict regulations on the number of characters allowed per tweet, making it an ideal platform for exploratory research. Nonetheless, the methodological approach in this study should be applicable to other channels as well since social media posts, in general, are short and unstructured (Kasperiuniene et al., 2020). However, it is still critical to note that the nature of social media differs in terms of user demographics, text presentation, or rhetoric, amongst others. Thus, future research should continue to explore the effectiveness of topic modeling algorithms across other platforms. Lastly, acknowledging the epistemological challenges of big data is also of importance; regardless of the massive volumes of data that may appear tempting at face value, algorithms should be contextualized in a particular social framework (Egger and Yu, 2022). Although topic models have quantified short-text social media data, both the interpretation and justification of the results come at the expense of data accuracy. Being equipped with extensive domain knowledge in data-driven science (Canali, 2016) would therefore allow social scientists to transform quantitative analytics into valuable insights for knowledge acquisition.

## Data Availability Statement

The original contributions presented in the study are included in the article/supplementary material, further inquiries can be directed to the corresponding author.

## Author Contributions

RE collected and analyzed the data. JY wrote the manuscript in consultation with RE and interpreted the data. Both authors designed the study and were responsible for the overall management and planning. All authors contributed to the article and approved the submitted version.

## Conflict of Interest

The authors declare that the research was conducted in the absence of any commercial or financial relationships that could be construed as a potential conflict of interest.

## Publisher's Note

All claims expressed in this article are solely those of the authors and do not necessarily represent those of their affiliated organizations, or those of the publisher, the editors and the reviewers. Any product that may be evaluated in this article, or claim that may be made by its manufacturer, is not guaranteed or endorsed by the publisher.
